# Evaluating the importance of metamorphism in the foundering of continental crust

**DOI:** 10.1038/s41598-017-13221-6

**Published:** 2017-10-12

**Authors:** Timothy Chapman, Geoffrey L. Clarke, Sandra Piazolo, Nathan R. Daczko

**Affiliations:** 1 School of Geosciences, The University of Sydney, Sydney, NSW 2006 Australia; 2 ARC Centre of Excellence for Core to Crust Fluid Systems and GEMOC, Department of Earth and Planetary Sciences, Macquarie University, Sydney, NSW 2109 Australia; 3 School of Earth and Environment, University of Leeds, Leeds, United Kingdom

## Abstract

The metamorphic conditions and mechanisms required to induce foundering in deep arc crust are assessed using an example of representative lower crust in SW New Zealand. Composite plutons of Cretaceous monzodiorite and gabbro were emplaced at ~1.2 and 1.8 GPa are parts of the Western Fiordland Orthogneiss (WFO); examples of the plutons are tectonically juxtaposed along a structure that excised ~25 km of crust. The 1.8 GPa Breaksea Orthogneiss includes suitably dense minor components (e.g. eclogite) capable of foundering at peak conditions. As the eclogite facies boundary has a positive *dP/dT*, cooling from supra-solidus conditions (*T* > 950 ºC) at high-*P* should be accompanied by omphacite and garnet growth. However, a high monzodioritic proportion and inefficient metamorphism in the Breaksea Orthogneiss resulted in its positive buoyancy and preservation. Metamorphic inefficiency and compositional relationships in the 1.2 GPa Malaspina Pluton meant it was never likely to have developed densities sufficiently high to founder. These relationships suggest that the deep arc crust must have primarily involved significant igneous accumulation of garnet–clinopyroxene (in proportions >75%). Crustal dismemberment with or without the development of extensional shear zones is proposed to have induced foundering of excised cumulate material at *P* > 1.2 GPa.

## Introduction

The foundering and removal of dense continental crust^1^ is part of the gross-scale geochemical and geodynamic cycling in magmatic arcs^2,3^. Foundering is predicted to occur in most arc systems^4^, though the short-lived nature of root detachment greatly restricts its direct observation via geophysical methods^2,5^. The re-incorporation of mafic crust into the mantle by foundering is interpreted to progressively contribute to refining the average composition of the continental crust to andesite^2,4^. Periodic crustal thickening, root removal and rebound during long-lived subduction can influence plate-scale cycles, including lithospheric contraction and extension^6^. Systematic overturn of the arc system through foundering can also stimulate magmatic flare-up^7^, linked to the melting of descending crust and the mantle^3,4^.

Foundering is a consequence of a gravitational instability when the lower crust develops a density greater than that of the underlying mantle (~3.33 gcm^−3^)^8–11^. The rheology of the crust and mantle are also important^8^. Typically, foundering of the base of the continent is considered to reflect either (1) Rayleigh-Taylor instabilities (“drip”) or (2) delamination, whereby modelling predicts that the base of the lithosphere peels away and sinks^2,12^. The exact mechanics of foundering in compositional stratified lower crust are not generally considered or known. The initial densification can occur by two main mechanisms. Igneous fractionation, involving the production of dense cumulate material, is thought to occur in most magmatic arcs^2,4^. Metamorphism can also increase the density of lower arc crust through: (i) its conversion to eclogite^9,11^; (ii) anatectic melt-extraction and the production of restite^13^; or (iii) large-scale metasomatic alteration^14^. The scarcity of deep arc exposures limits our understanding of the relative importance of these mechanisms.

We present a case study of well-exposed deep arc crust to evaluate the efficiency of closed-system metamorphism to produce crust that is denser than the upper mantle. Eclogite, with or without high-*P* granulite, is predicted to be a consequence of crustal thickening, dependent on lithospheric heat flow^15,16^ and composition. The conversion of crustal rocks to eclogite has been well studied through direct experimentation^15^ and the investigation of natural examples^9,17^. These studies assume the lower crust is primarily of basic (gabbroic) composition, but an intermediate (dioritic) composition may be more appropriate^18,19^. Studies of exposed lower crust also indicate that the metamorphic conversion of gabbro to eclogite is commonly incomplete, with the scale and extent of equilibration being dependent on fluid abundance and strain intensity^17,20^. Protolith composition and the efficiency of metamorphic conversion are fundamental in producing rock sufficiently dense to founder. However, complexity comes from the crust being compositionally heterogeneous^18,19^, and the longer time scale of metamorphism enabling its potential to overlap with significant contractional and/or extensional deformation^6,20^. The mechanical dynamics of density sorting and inversion may thus potentially be more complicated than simple root slumping.

**Figure 1:**
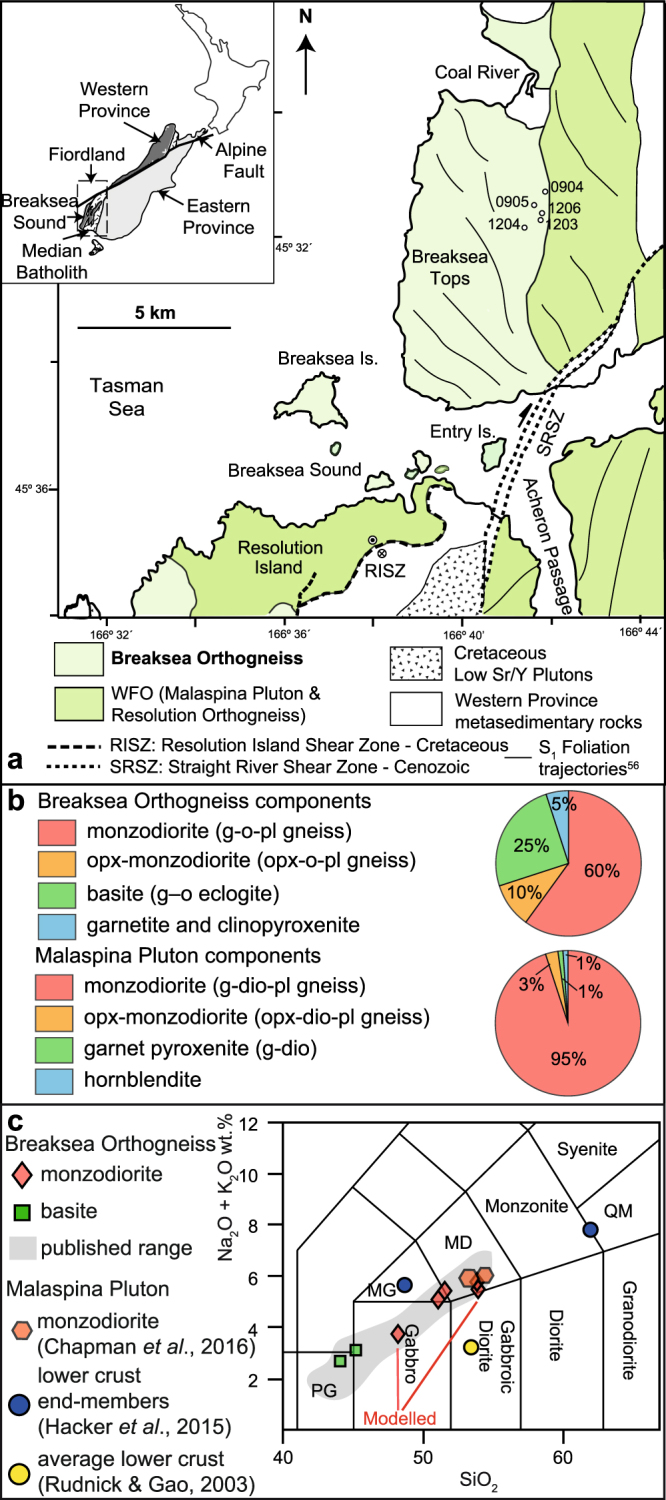
(**a**) Simplified geological map of the Breaksea Sound area, between northern Resolution Island and Coal River. Cretaceous plutons intrude Palaeozoic metasedimentary rocks or are separated by shear zones. Two mappable units of the WFO are shown; the Breaksea Orthogneiss and Malaspina Pluton. Circles show sample locations used in this study and the lines represent S_1_ fabric trajectories^31,56^. The map is modified from Turnbull *et al*.^56^ and the sample locations were prepared using QGIS (http://www.qgis.org/), both available under CC BY 4.0 license (https://creativecommons.org/licenses/by/4.0/). See^31,36^ for detailed cross-sections across the area. (**b**) Proportions of components that makeup the Breaksea Orthogneiss and Malaspina Pluton. (**c**) Total–alkali silica diagram^45^ displaying the variation in whole-rock compositions of the Breaksea Orthogneiss and Malaspina Pluton (shadow). Predicted bulk compositions^18,19^ of the lower crust are shown for comparison. Abbreviations include: peridotgabbro (PG), monzodiorite (MD), monzogabbro (MG), quartz monzonite (QM).

This study evaluates eclogite and high-*P* granulite formed along the Cretaceous Proto-Pacific Gondwana margin, and exposed in SW Fiordland, New Zealand. Exposures of the Western Fiordland Orthogneiss (WFO) mostly reflect two major structural levels (1.2 and 1.8 GPa), and examples of the two levels are tectonically juxtaposed^21,22^. The high-*P* Breaksea Orthogneiss was a composite layered pluton of monzodiorite and gabbro that was patchily deformed and recrystallised at ~850 °C and 1.8 GPa^21,23^. It experienced limited partial melting during metamorphism, as it lacks peritectic phase assemblages and maintains incompatible-element-rich whole-rock compositions^21^. The Malaspina Pluton (Fig. 1) presents a shallower equivalent of the Breaksea Orthogneiss^22^. In SW Fiordland, it is largely garnet monzodioritic gneiss with discontinuous layers of garnet pyroxenite, that reflect limited partial melting, patchy deformation and recrystallization at ~750 °C and 1.2 GPa^24^. These WFO plutons present a striking natural laboratory with rock compositions^18,19^ appropriate to evaluate the importance of metamorphic densification at *P–T* conditions commonly posited for lower crustal foundering. The preservation of the tectonically disrupted lower crustal sequence begs explanation as to the mechanisms that could lead to density sorting and foundering. Our results resolve the *PT* conditions and compositions that will produce arc crust denser than upper mantle rocks. Two models are then evaluated in the context of observed field relationships: foundering being linked to crustal extension, or counter-flow diapirism.

## Geological setting and previous work

Fiordland, New Zealand exposes Late Cretaceous rocks from a Cordilleran-style magmatic arc developed on the Pacific Gondwanan margin (Zealandia)^25^. The arc batholith is dominated by the high Sr/Y *c*. 126–105 Ma Separation Point Suite and Western Fiordland Orthogneiss (WFO)^22,26–30^, which locally intrude Early Palaeozoic continental metasedimentary rocks and separate them from accreted Permian–Cretaceous volcano-sedimentary terranes^25^. The WFO comprises a series of intermediate to mafic plutons emplaced inboard of the continental margin at various depths (0.9–1.8 GPa)^22^. The exposed WFO sequence is tectonically disrupted, with plutons emplaced at 1.2 GPa now structurally juxtaposed with plutons emplaced at 1.8 GPa^22,29^. The WFO is dominated by gabbroic to monzodioritic protoliths, with the presence of distinct Na-Ca clinopyroxenes reflecting different emplacement depths^21,22,24,31^. All WFO plutons have HREE-depleted bulk rock and mineral compositions, consistent with their formation having involved substantial garnet fractionation^22,24,28,32^. The Breaksea Orthogneiss presents the highest grade rocks (Fig. 1a), including omphacite granulite and eclogite (*T* ≈ 850 °C and *P* ≈ 1.8 GPa)^21,33^. It is composite (Fig. 1b), being formed mostly of monzodioritic gneiss (~60–65%) with cognate orthopyroxene-bearing portions (~5–10%), and cumulate basite (now eclogite ~25%), clinopyroxenite, garnetite and harzburgite (~5%)^21,23,34^. The extensional Resolution Island Shear zone forms an upper carapace to the Breaksea Orthogneiss, juxtaposing it with the Malaspina Pluton (1.0–1.2 GPa) and Palaeozoic metasedimentary rocks at *c*. 105–90 Ma^21,35^. The Malaspina Pluton represents a voluminous magma body emplaced and patchily recrystallized at ~750 °C and 1.2 GPa^24,29^. It comprises garnet monzodioritic gneiss (~95%: Fig. 1b) with cognate two-pyroxene monzodioritic gneiss (~3%), discontinuous layers of garnet pyroxenite (~1%) and hornblendite (~1%)^24^. More pronounced HREE depletion of the Malaspina Pluton relative to the Breaksea Orthogneiss is consistent with the loss of appreciable garnet residue from the exposed sequence, most likely in the pressure range of 1.3–1.7 GPa^24^. In this paper, we primarily evaluate the capacity for the Breaksea Orthogneiss to founder as it reflects the highest-*P* conditions, although many relationships are similar for the Malaspina Pluton.

The Breaksea Orthogneiss records incomplete metamorphism and patchy deformation (D_1_) that occured during cooling at high-pressure conditions (1.8–2.0 GPa) subsequent to its plutonic emplacement^21^. Post-D_1_ decompression to *P* ≈ 1.0–1.4 GPa and *T* ≈ 650–750 °C is recorded by diopside-albite symplectite^21^ that partially to completely pseudomorphs omphacite and its inferred to correspond to a stage of gneiss dome formation^35,36^. Upper amphibolite facies assemblages developed locally in D_2_ shear zones that are thought to be related to orogenic collapse^29,35^. This work explores outcrops at Breaksea Tops away from the effects of the major D_2_ shear zones (Fig. 1a). In accordance with the persistence of protolith relicts in the orthogneiss, terminology specific to both igneous and metamorphic stages is used to clarify inferred processes.

### Previous work

Basic and monzodioritic, now eclogite and granulite, gneiss of the Breaksea Orthogneiss preserve gradational contacts in outcrop and linear first-order trends in Harker plots of whole-rock composition (Fig. 1c)^21^. Variations in the proportions of garnet, clinopyroxene, orthopyroxene and plagioclase in the orthogneiss are mostly attributed to cumulate processes and magma redox conditions that preceded high-grade D_1_ deformation^23,34–36^. Interlayered near-monomineralic garnetite and clinopyroxenite retain delicate microstructure and mineral chemistry consistent with an igneous origin^23^. Garnet in basite components can also interpreted to be of igneous origin because its major and rare earth element (REE) characteristics are similar throughout the spectrum of ultrabasic to intermediate compositions and chondrite-normalised REE patterns lack positive Eu anomalies^23^. As garnet and omphacite collectively form 90% of basite layers, this is also then consistent with most omphacite in the eclogite layers being of cumulus origin^23^.

Similar igneous mineralogy is observed in monzodioritic gneiss that is interlayered with the basite, garnetite and clinopyroxenite; mineral REE concentrations overlap among all rock types. Igneous and metamorphic garnet are distinguished in the monzodioritic gneiss by microstructural and chemical features: (1) large euhedral garnet grains in clusters with omphacite retain heavy-REE-enriched patterns that overlap with those of igneous garnet in eclogite and garnetite; and (2) idioblastic garnet grains forming coronae that separate omphacite and plagioclase are heavy-REE depleted and have positive Eu anomalies consistent with metamorphic growth during consumption of plagioclase. In places, foliated (S_1_) assemblages of omphacite, garnet, plagioclase, kyanite and rutile are consistent with metamorphic equilibration at conditions of the omphacite granulite sub-facies^34^. The nature of the S_1_ fabrics varies between exposures in the mouth of Breaksea Sound and those at Breaksea Tops^33^. Outcrop relationships at Breaksea Sound preserve S_1_ with both vertical and near horizontal orientations that define concentric, decimetre-scale gneiss domes spatially related to large extensional D_2_ shear zones^35,36^. These dome structures are not observed at Breaksea Tops. In addition, the fabric trajectories are oblique to the unit boundaries, inconsistent with gross-scale diapiric flow (Fig. 1a).

## Results

### Field Relationships

Garnet, clinopyroxene and plagioclase, with or without orthopyroxene, K-feldspar, quartz and kyanite, form monzodioritic gneiss that makes up much of the Breaksea Orthogneiss (Fig. 1b). There are both gradational and sharp contacts between layers and cumulate pods of basite, garnetite and clinopyroxenite. An igneous layering at Breaksea Tops is locally cut by a moderately dipping (~65°), north-striking gneissic foliation (S_1_) with an associated L_1_ mineral stretching lineation plunging towards the southeast (Fig. 1a). Foliation trajectories are consistent with only local deviations in strain shadows to basite pods (Fig. 1a). Whole rock major and trace element compositions throughout the gneiss are remarkably consistent, defining a restricted range in silica and total alkali content between monzogabbro and monzodiorite (SiO_2_ = 48–55 wt.% and Na_2_O + K_2_O = 4–7 wt.%) (Fig. 1c)^23^. The bulk composition of the gneisses resembles those predicted for the lower crust (Table 1)^18,19^.

**Table 1:** Whole-rock compositions of the granulite in the Breaksea Orthogneiss and predictions of the lower crust^18,19^.

Sample	BS0905B	BS1203C	BS1206	Allibone *et al*.^22^ P73824	Rudnick & Gao^19^	Hacker *et al*.^18^	
						mafic	felsic
SiO_2_	51.10	53.58	53.88	48.08	53.40	48.60	61.90
TiO_2_	1.06	1.07	1.07	1.59	0.82	1.40	0.78
Al_2_O_3_	18.78	18.00	18.38	16.93	16.90	18.10	16.10
Fe_2_O_3_	9.83	8.62	8.06	11.70	7.71	9.39	5.87
MnO	0.18	0.14	0.13	0.15	0.10	0.18	0.11
MgO	5.31	4.82	4.60	7.15	7.24	6.87	3.14
CaO	7.99	7.79	7.75	9.30	9.59	10.11	5.77
Na_2_O	4.12	4.30	4.41	3.28	2.65	2.85	3.92
K_2_O	0.99	1.21	1.38	0.49	0.61	2.85	3.92
P_2_O_5_	0.41	0.37	0.33	0.65	0.10	0.23	0.21
LOI	0.18	0.10	−0.04	0.31			
**Total**	**99.77**	**99.90**	**100.02**	**99.63**	**99.12**	**100.58**	**101.72**

LOI, loss on ignition.

### Petrography

**Figure 2:**
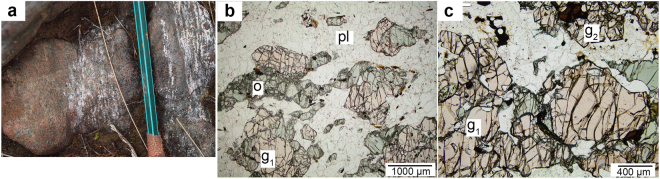
(**a**) Outcrop photograph of the interlayered monzodioritic gneiss and garnet–omphacite cumulate of the Breaksea Orthogneiss. (**b**) Photomicrograph of low-strain monzodioritic gneiss comprising intergrowths of euhedral omphacite and Type 1 garnet (g_1_). (**b**) Photomicrograph of monzodioritic gneiss of the Malaspina Pluton comprising diopside, Type 1 and Type 2 garnet (g_2_) coronae. Samples of the Malaspina Pluton are nearly identical in textures and modes^24^.

Mineral assemblages are subtly distinct across the compositional spectrum of the Breaksea Orthogneiss, although textural relationships remain largely similar. Monzodioritic gneiss comprises varying proportions of plagioclase, omphacite, orthopyroxene, igneous (Type 1) and metamorphic (Type 2) garnet, rutile, K-feldspar, quartz, kyanite, ulvöspinel, hornblende and apatite. Large (300–1000 μm) Type 1 garnet and omphacite grains form mm-scale grain clusters in monzodioritic gneiss, that are variably elongated (Fig. 2b). Grain cores of Type 1 garnet have rutile exsolution lamellae and euhedral inclusions of feldspar and omphacite. The facetted feldspar inclusions are generally antiperthite or comprise composite inclusions of plagioclase surrounded by narrow K-feldspar rims. Exsolution-free rims to the Type 1 garnet cores have rounded omphacite and plagioclase inclusions. Omphacite may locally preserve euhedral shapes (Fig. 2b) and have facetted plagioclase inclusions and/or fine oxide exsolution lamellae in grain cores. Some portions of the gneiss contain coarse orthopyroxene (800–1000 μm) intergrown with omphacite and ulvöspinel, with only minor Type 1 garnet. In sites of higher strain, Type 2 garnet coronae separate elongated omphacite from plagioclase. Quartz, K-feldspar and rutile are intergrown with Type 2 garnet, commonly as vermicular inclusions (Fig. 2c). Type 1 garnet is absent or occurs in low modes in areas of Type 2 garnet abundance. The coarse-grained feldspar-rich matrix enclosing the garnet–omphacite clusters is dominated by plagioclase, with minor K-feldspar, quartz, kyanite, garnet and omphacite (Fig. 2b). K-feldspar occurs interstitial to plagioclase with minor quartz. Kyanite occurs as tabulate and acicular grains, aligned within S_1_ in the feldspar-rich matrix of high-strain samples.

**Table 2:** Temperature estimates for stages of the Breaksea Orthogneiss high-pressure evolution.

Sample	Assemblage	Assumed	Calculated	±	*n*	Method
		*P* (GPa)	*T* (°C)			
1206	g_1_	1.8	1022	50	9	Ti-in-g^39^
1204J2	g_1_	1.8	920	30	9	Zr-in-ru^53^
1203 T	g_1_	1.8	950	50	9	Ternary Fsp^54,55^
1204J2	g_2_ necklace	1.8	845	30	12	Zr-in-ru^53^

Decimetre-scale cumulate basite comprises near-equal proportions of garnet (52%) and omphacite (40%) with accessory hornblende (4%), rutile (2%), plagioclase (1%) and apatite (1%) (Fig. 2a). Orthopyroxene is present in some pods in modal abundances of up to 15%^28^. A prominent layering involving variations in garnet and omphacite mode is cut by an S_1_–L_1_ fabric. Coarse (1000–2000 μm) garnet is equant and euhedral, with rutile exsolution lamellae and tabulate omphacite inclusions restricted to grain cores. Omphacite (700–1000 μm) has equant to elongated grain shape and is intergrown with garnet. In places, omphacite preserves either oxide or rutile exsolution lamellae in grain cores. Fine-grained hornblende (<200 μm) surrounds omphacite and garnet.

### Mineral chemistry

Omphacite grain cores have lower jadeite content (Jd_26__–__27_ = 100[(2Na/(2Na + Ca + Mg + Fe^2+^)) (Al_M1_/(Al_M1_ + Fe^3+^_M1_))]) than grain rims (Jd_30__–__31_). Omphacite inclusions in Type 1 garnet have the lowest jadeite contents (Jd_12__–__14_). Variations in the granulite bulk rock composition have a minor effect (5% variation) on the jadeite content.

Type 1 garnet grains show core to rim zoning in grossular content (100[Ca/(Fe^2+^ + Mn + Mg + Ca)]), with grain cores of Grs_6–8_ enclosed by comparatively enriched rims of Grs_8__–__13_. The grain cores of Type 1 garnet have elevated TiO_2_ concentrations (0.11–0.15 wt.%) compared with rims (0.03–0.05 wt.%). Type 2 garnet compositions broadly match that of garnet rims (Grs_12__–__18_).

Homogeneous plagioclase inclusions in Type 1 garnet have the highest anorthite content (An_29–35_ = 100[Ca/(Ca + Na + K)])) in a range between An_22_ and An_35_. The inclusions are commonly oligoclase (An_16–18_) with orthoclase exsolution lamellae (Or_78–91_ = 100[K/ (Ca + Na + K)]). Matrix plagioclase is mostly andesine (An_28–35_) with grains adjacent to Type 2 garnet having low anorthite contents (An_23–25_). Orthoclase (Or_87–88_) occurs intergrown with Type 2 garnet.

The zirconium concentrations of rutile inclusions in Type 1 garnet lie between 2600 to 6000 ppm. Rutile intergrown with Type 2 garnet in necklace textures has less zirconium (1800–2400 ppm).

### P–T path and Mineral Equilibria Modelling

**Figure 3:**
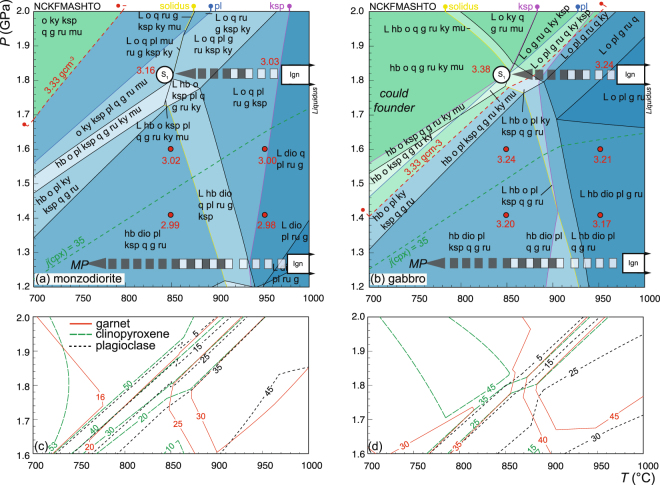
Pressure–temperature (*P–T*) pseudosections constructed in the NCKFMASHTO system using THERMOCALC for a monzodiorite (**a**) and gabbro composition (**b**). Different shading of the fields represents changes in variance. The key mineral-out boundaries and location of the omphacite–diopside transition (*j*(cpx)* = *100[Na/(Na + Ca)]) are marked. The density threshold for foundering (3.33 gcm^−3^) is highlighted by the dashed red line, above which fields have green shading, small labelled red circles represent specific density outputs. The inferred high-pressure cooling path from sub-liquidus igneous (Ign) to S_1_ conditions is displayed based on observed modes and petrological relationships in the Breaksea Orthogneiss. An additional path for the Malaspina Pluton (*MP*) is highlighted for comparison of the two units. (**c–d**) Modal isopleths for garnet, clinopyroxene and plagioclase shown for key regions of interest from (**a**,**b**). Mineral abbreviations outlined in the methods section.

Pressure–temperature pseudosections (*P–T*) are presented (Fig. 3) for monzodioritic and gabbroic compositions observed in the Breaksea Orthogneiss (Fig. 1c). The pseudosections indicate the dependence of mineral assemblage on changes in pressure, temperature and composition^37,38^. The modelled conditions encompass the granulite – eclogite transition for gabbroic and monzodioritic protoliths (Table 1). The upper pressure stability of plagioclase marks the lower boundary of the eclogite facies (Fig. 3a,b). The main effect of lowering the bulk-rock silica content from a monzodiorite to a basite protolith is that the eclogite facies boundary is encountered at lower pressure conditions (1.9 *cf*. 1.8 GPa: Fig. 3a,b)^33^. Gabbroic compositions have less feldspar (plagioclase and K-feldspar) and quartz, and higher modes of garnet and hornblende at the expense of omphacite when contrasted with monzodioritic compositions at the eclogite facies boundary (Fig. 3).

Mineral assemblage and texture define two high-*P* stages in the monzodioritic gneiss of the Breaksea Orthogneiss. Granoblastic, HREE-enriched Type 1 garnet intergrown with coarse omphacite is interpreted as igneous (Fig. 2b)^23^. Hypersolvus feldspar and high-Zr rutile inclusions in Type 1 garnet reflect *T* > 950 °C (Table 2 and Fig. S1). The high-Ti content, and rutile exsolution in Type 1 garnet potentially extends these estimates to above 1000 °C^39^. Part of the igneous equilibria are likely accounted for by the quinivariant field (L–o–g–q–pl–ru) for monzodioritic compositions (Fig. 3a). The igneous assemblages are partially recrystallized by metamorphic reaction textures involving Type 2 garnet intergrown with jadeitic omphacite, albitic plagioclase, quartz and rutile (Fig. 2c). Metamorphic mineral textures are consistent with recrystallization both accompanying the development of S_1_ and post-dating it. Zirconium-in-rutile thermometry from Type 2 grains within necklace textures suggests growth at *T* ≈ 850 °C, and match other mineral exchange estimates for the peak metamorphic conditions (Table 2)^21^. The quadrivariant field (o–g–ksp–pl–ru–q–ky–mu) best accounts for the mineral modes and S_1_ textures (Fig. 3a)^33^. Feldspar and garnet exsolution textures and mode changes, including a reduction in anorthitic plagioclase and the growth of jadeitic omphacite, in the monzodioritic gneiss are best explained by a high-*P* cooling history. Key high-variance changes induced by isobaric cooling at 1.8 GPa mostly involve the breakdown of anorthitic plagioclase and growth of omphacite. Garnet mode initially increases with cooling to ~900 °C, before decreasing with further cooling to accommodate Fe-Mg exchange related to omphacite mode increases at lower *T*. Equivalent changes could be induced by a *PT* path involving pressure increasing from ~1.5 to 2.0 GPa, but such a path is predicted to be associated with larger changes in the type and composition of the Ca-Na clinopyroxene than are observed. An equivalent isobaric cooling *PT* path at lower pressure conditions (<1.5 GPa) in a composite pluton of gabbroic or monzodioritic composition for the Malaspina Pluton would encompass an equivalent series of reactions involving diopside instead of omphacite and higher plagioclase modes (Fig. 3)^24^.

### Measured and Calculated Rock Density

**Figure 4:**
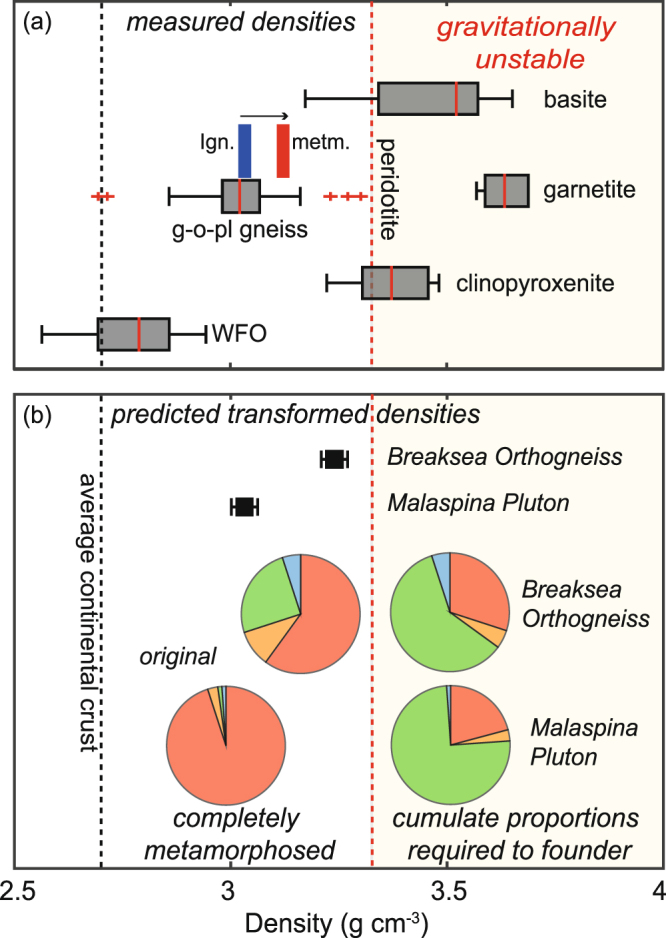
Prediction of gravitational instability (compared to mantle peridotite) based on dry density determinations of natural samples (**a**). Box represent interquartile range from the median band in red; whiskers reflect 1^st^ and 99^th^ percentiles and red plusses are outliers. (**b**) predicted densities (black squares) based on measured interlayered Breaksea Orthogneiss lithologies and Malaspina Pluton^24^. Pie-graphs display observed lithology proportions (Fig. 1b; orange = orthopyroxene monzodiorite, blue = garnetite/clinopyroxenite or hornblendite, red = garnet monzodiorite and green = garnet–clinopyroxene basite) and predicted volumes appropriate for gravitational instability (right). Error bar on densities based on predicted bulk modal variation of ~5%.

Measured rock densities increase from samples with predominately igneous textures (3.02 to 3.03 gcm^−3^) to those that are dominated by metamorphic textures (3.12 gcm^−3^) (Fig. 4a). The data range overlaps with previous measurements made of WFO samples (2.6–3.15 gcm^−3^)^33^. The theoretical density of a completely metamorphosed Breaksea Orthogneiss can be calculated as 3.24 gcm^−3^ by using the observed proportions of its main components, involving 30% garnet-omphacite basite (both eclogite and monomineralic layers) at 3.45 gcm^−3^ and 70% garnet-omphacite granulite (converted garnet- and orthopyroxene-monzodiorite) at 3.15 gcm^−3^ (Fig. 4b). The proportion of cumulate basite within the orthogneiss would need to exceed 60% by volume for the Breaksea Orthogneiss to be capable of foundering (Fig. 4). As the Malaspina Pluton has higher feldspar modes (~3.00 gcm^−3^), the proportion of cumulate basite (3.45 gcm^−3^) at 1.3–1.7 GPa must exceed 75% at 1.2 GPa to result in arc crust with the potential to founder (Fig. 4b).

Rock densities calculated from mineral equilibria modelling of the granulite – eclogite transition have trends dependent on both composition and *PT* conditions (Fig. 3a,b). In general, higher pressure conditions induce higher densities in both of the compositions modelled, but the most pronounced changes are associated with the destabilisation of plagioclase at the lower boundary of the eclogite facies. As key mineral assemblages associated with this change have boundaries with high *dP*/*dT* values, density should also increase during recrystallization on a *PT* path involving cooling (e.g. 950 to 850 °C) at constant pressure (Fig. 3a,b), assuming that the rocks fully equilibrate. More basic compositions have densities ~0.22 gcm^−3^ higher than intermediate compositions at equivalent *P–T* conditions (Fig. 3). Assuming a density of 3.33 gcm^−3^ for the upper mantle, monzodioritic gneiss will be capable of foundering at a pressure 0.15–0.2 GPa above the granulite – eclogite transition, whereas basic gneiss may founder at ~0.5 GPa below the transition (Fig. 3). Conditions critical to foundering are thus not strictly dependent on the eclogite facies boundary or garnet production. The higher pressure and lower temperature changes in monzodioritic gneiss density are mostly associated with K-feldspar stability and omphacite mode (Fig. 3a). In basic gneiss, the higher garnet and omphacite modes give higher rock densities despite the presence of plagioclase and melt stability (below the eclogite facies, Fig. 3b).

## Discussion

The veracity and scale of processes plausibly resulting in, and derivative from a gravitational instability remain controversial, and are complicated by the continental crust being heterogeneous^2,3^. Using the case study of the Zealandia magmatic arc, we resolve the blend of intrinsic and extrinsic parameters that have the potential to facilitate the foundering of the lower continental crust, and contrast this detail with macroscopic features presented by the case study.

### How to founder the lower crust

To induce foundering of the lower crust, geological processes need to form rock of density higher than the majority of the continental crust and the upper mantle^8^. The key minerals required to produce such high densities in common crustal rocks are garnet (>3.6 gcm^−3^) and omphacite (~3.16–3.4 gcm^−3^), their co-occurrence characterising the eclogite facies^15^. The high-variance reactions delineating the granulite – eclogite transition are controlled by plagioclase breakdown^15,33^, which will be accompanied by a density step in the profile of basic or felsic lower crust. However, at high-*T* conditions much of the change is controlled by high variance pressure-dependent reactions in granulite: the progressive consumption of the albite component in plagioclase facilitates the growth of omphacite via solid solution, involving the substitution of the Ca-Tschermak’s and jadeite molecule^15^. The substitution is coupled with the consumption of grossular-rich garnet through Fe–Mg exchange with omphacite^15^. However, the positive *dP/dT* of the relevant high-variance reactions means that near isobaric cooling can also lead to eclogite formation (Fig. 3).

Metamorphism of the Breaksea Orthogneiss incompletely records cooling from high-*T* conditions associated with igneous crystallization, metamorphic minerals being distinguished by sodic plagioclase, and marked changes in the jadeite content of clinopyroxene and grossular content of Type 2 garnet. Igneous garnet, clinopyroxene and hypersolvus feldspar record high igneous crystallization temperatures (>950 °C) in grains that were patchily metamorphosed and deformed at lower-temperature conditions (~850 °C). The patchy metamorphism is marked by the development of comparatively dense metamorphic mineral assemblages (Figs 3 and 4). Similar, though less pronounced densification is predicted for igneous rocks cooling at lower pressure conditions, in the Malaspina Pluton (Fig. 3)^24^.

The eclogite facies boundary is commonly considered a minimum depth to form rock of density adequate to induce crustal foundering^15,18^. However, the upper pressure limit of plagioclase is sensitive to changes in bulk-rock composition and is predicted to occur at lower pressure conditions in more basic compositions (Fig. 3)^15,33^. In addition, rocks that recrystallized in the eclogite facies need not have densities exceeding that of the upper mantle. The densities of high-*P* intermediate and basic gneiss are mostly dependant on omphacite and garnet mode. The proportions of both these minerals are in turn dependent on pressure and temperature conditions, as can be shown by comparing the different *PT* conditions required for gabbroic and monzodioritic protoliths to have densities above that of the upper mantle (3.33 gcm^−3^). Metamorphosed gabbroic protoliths are predicted to have densities above 3.33 gcm^−3^ in plagioclase-bearing equilibria, even at suprasolidus (melt-present) conditions (Fig. 3b). In contrast, metamorphosed monzodioritic protoliths are predicted to have densities less than 3.33 gcm^−3^ until the progressive breakdown of K-feldspar at T ≈ 750 °C, some 0.1–0.2 GPa beyond the granulite – eclogite transition (Fig. 3a). Mineral mode changes modelled across the transitions are marked mostly by plagioclase consumption and omphacite growth. Garnet may be either a product or a reactant (Fig. 3). The densification of intermediate to basic rocks is therefore not as dependant on garnet growth as commonly considered^15,18^. Basic compositions have greater capacity to develop higher garnet modes that attribute higher densities.

Mineral assemblage changes associated with a given rock moving from the granulite to eclogite facies do not always run to completion^38^. The metastable persistence of porphyroclastic or phenocrystal material at elevated metamorphic temperature conditions (e.g. >750 °C) is not generally explicitly considered but seems quite common^10,17,20^. For this case study, the efficiency of metamorphism in the Breaksea Orthogneiss would seem to have been limited by a comparatively short timescale (*c*. 10 Myr), heterogeneity in strain and restricted fluid/melt availability^23,30,31,34^. The high proportions of relict minerals would have enhanced buoyancy of the lower arc crust, and reduced its chance of foundering (Fig. 4).

The average silica content of the lower continental crust is generally thought to be above 50 wt.% (Table 1)^18,19^ presenting difficulties for closed-system metamorphism to induce a gravitational instability at common crustal thicknesses. Mechanisms that will be more effectual in producing dense lower crust include: (i) igneous accumulation; (ii) partial melt extraction; and (iii) large-scale metasomatism. Varying combinations of basic cumulate and intermediate rocky types are observed in most deep arc systems^2,18^, their formation related to arc melting. The presence of garnet–clinopyroxene basite in both the Breaksea Orthogneiss^23^ and Malaspina Pluton highlights the potential for igneous cumulates to densify the deep crust in arc systems, all cumulate compositions having densities higher than mantle peridotite (Fig. 4). However, the potential for the Fiordland basite to founder is dependent on its proportion relative to the interlayered, buoyant monzodioritic gneiss (Fig. 4). The proportion of garnet–clinopyroxene cumulate in the Breaksea Orthogneiss would need to exceed 60% to reach densities appropriate for its foundering (Fig. 4). In thinner arc systems (~40 km), the proportions of cumulate would potentially need to be even greater (above 75%) on account of higher plagioclase mode (Fig. 4)^24^.

### Foundering mechanisms: crustal dismemberment

The continental crust in most of Earth’s magmatic arcs is approximately 40 km thick (~1.2 GPa at its base)^40^, with Cordillera Zealandia having been at least 65 km thick (~1.8 GPa) and probably thicker^21^. Primitive crustal material similar to, or more basic than, the Breaksea Orthogneiss is inferred to underlie Fiordland based on seismic velocity models^41^. This would extend arc crust to depths greater than 80 km^7,32^. The presence of such material underneath the Breaksea Orthogneiss is supported by its depleted Sr/Y and REE ratios and Hafnium isotopic signatures^7,24^. Few examples of such thick arc crust are evident in the geological record, plausibly because of root detachment^40,42^. Rock densities of both igneous and metamorphic stages in the Breaksea Orthogneiss are too low to potentially induce foundering, mostly due to its composition. It seems reasonable to assume that the overall composition of the seismically-imaged material is similar, as it also did not founder^41^. The foundering of any monzodiorite-dominated crust thus seems negligible, but a significant proportion of the Cretaceous arc crust between 1.2 and 1.8 GPa has not be identified and is missing. In addition, a large proportion of residual material (~25% by volume) complementary to felsic high Sr/Y batholiths that accumulated between 1.2 and 1.0 GPa is not recognized in the Fiordland section^24^. Theas features are consistent with material having been lost from the Fiordland arc, but perhaps not from the level represented by the Breaksea Orthogneiss (1.8 GPa).

**Figure 5:**
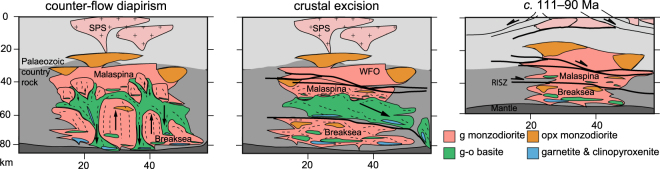
Crustal schematic of the possible evolution of the Zealandia magmatic arc through the period *c*. 120–105 Ma (figure adapted from Klepeis *et al*.^43^). Possible foundering mechanisms involving diapiric counter-flow^36^ or crustal excision (this study).

Two main mechanisms can be envisaged for density sorting above the arc base: (1) diapirism and counter-flow related to the concentric dome fabrics present in the WFO^36^; or (2) crustal excision along extension shear zones (Fig. 5). Density sorting via counter-flow diapirism is difficult to reconcile in Fiordland on account of: (i) the 100 m to km-scale of the domes^36^ being 1 to 2 orders of magnitude too small to have controlled appreciable foundering; (ii) the patterns are developed in material that remained positively buoyant (even for eclogite); (iii) the domes in Breaksea Orthogneiss at Breaksea Sound likely formed after its decompression to ~1.2 GPa (albite-diopside symplectites)^21^ and are spatially linked to the RISZ (Fig. 1a); (iv) gross scale counter-flow pathways are not observed at the deeper levels recorded in the Breaksea Orthogneiss at Breaksea tops (Fig. 5). It is possible that the dome patterns reflect a larger process that completely removed dense material elsewhere^36^, but that then begs the question of how to make the dense material. At the conditions attributed to dome formation (~1.2 GPa), even pure basite would need to cool and recrystallize below *T* ≈ 600 °C to be negatively buoyant relatively to the upper mantle (Fig. 3b) and there would need to be a lot more of it than is observed.

Igneous processes are the most viable mechanism to produce a crust with a high-density layer of cumulate material. The composition of WFO plutons that accumulated at *P* < 1.8 GPa indicate that significant crystal fractionation or crustal mixing must have occurred, leaving a dense residue^24,30,32^. The large volume of HREE-depleted WFO magma compositions that accumulated at depths between 1.0 and 1.2 GPa, together with observed garnet–clinopyroxene cumulates, supports the concept of a cumulate pile having underlain this part of the arc crust^22,24^. We posit that the missing section of the crust comprised up to 75% garnet–clinopyroxene basite to account for the high Sr/Y ratios in the Malaspina Pluton^2,24^.

The posited cumulate material would have overlain more buoyant material (Breaksea Orthogneiss), creating a crustal sequence that is distinct to most generalized predictions of arc lithosphere^18,19,42^. As diapiric counter-flow would seem to have been incapable of density sorting in the Cretaceous Zealandia arc, it is proposed that crustal excision was a driver. Large-scale shear zones present an ideal mechanism to facilitate the excision of dense material from the crust and the RISZ juxtaposes 1.2 and 1.8 GPa components presented by the Malaspina Pluton and the Breaksea Orthogneiss^35^. Extensive conjugate shear zones record *c*. 111 Ma displacements of up to 9–25 km^29,36,43^ that are localized within, and adjacent to, WFO plutons^22^. Dense cumulate portions in the section between 40 and 65 km (Fig. 4), could have feasibly migrated both laterally and vertically along active shear zones during the period of fault activity^31,36^. The hanging wall material would have been suitably located to subsequently founder into the mantle^12^. The architecture could also possibly induce the return flow of depleted mantle material, to account for harzburgite xenoliths in the Breaksea Orthogneiss^21,44^. In addition to contribution to decimetre-scale dome perturbations in the WFO^36^. If such a crustal dismemberment mechanism eventuated, the Zealandia system involved foundering during extension at the end-stages of a magmatic flare-up cycle^3,6^. Extension could therefore be a viable period for crustal destruction and the initiator of a new Cordillera tectonic cycle^6^.

## Methods

Crushed whole-rock powders were analysed by X-ray fluorescence (XRF) spectrometry on a PANalytical PW2400 at the University of New South Wales for major element compositions. Protolith classification (Fig. 1c) followed Middlemost^45^. Mineral chemistry was determined on a CAMEBAX SX100 electron microprobe (Macquarie Geoanalytical) with a 15 kV accelerating voltage and a beam current of 20 nA. Zr-in-rutile analyses were performed on a Agilent 7700cs Inductively Coupled Plasma Mass Spectrometer (ICPMS), with attached 213 nm Nd:Yag laser ablation microprobe (LAM). Operating conditions involved a *c*. 60 s background period count prior to laser ablation, and a *c*. 100–120 s analysis using a 50 μm beam diameter and 5 Hz pulse repetition rate. The analysis of NIST 610 glass during each session provided an external standard and standard reference material BCR2, was analysed as an internal standard. Precision was 1% relative standard deviation, based on 1σ distribution on Zr.

Phase equilibria modelling was performed in the NCKFMASHTO chemical system (Na_2_O–CaO–K_2_O–FeO–MgO–Al_2_O_3_–SiO_2_–H_2_O–TiO_2_–O) using THERMOCALC version 3.45i^37^ and the internally consistent thermodynamic dataset 6.2 (updated 6^th^ February 2012)^46^. Mineral activity–composition models and abbreviations used include tonalite melt (L), hornblende (hb), omphacite/diopside (o/dio)^47^, feldspars (pl & kfs)^48^, garnet (g), muscovite (mu)^49^ and ilmenite (ilm)^50^. Pure phases include rutile (ru), quartz (q), kyanite (ky) and H_2_O. The modelled bulk rock compositions are based on XRF analysis of a monzodiorite (1203 C) and a non-cumulate gabbro (P73824^22^: Fig. 1c). The modelled redox conditions (Fe^3+^/[Fe^3+^/Fe^2+^] = 0.18) are generally considered appropriate for arc magmas^51^. Modelled water content (1 wt.%) is based on the observed modal proportions of hydrous phases (hornblende). The mineral chemistry is model dependent and shows partial differences to the observed compositions, although all changes match relative variations. Pressure uncertainties for assemblage field boundaries are approximately ± 0.1 GPa at the 2σ level^52^. The use of sodic-calcic pyroxene at suprasolidus conditions produced limited difference in field boundaries compared to the calibrated augite model^47^, on account of the low melt modes. It was utilised due to the importance of Na–Ca exchange.

Zirconium-in-rutile thermometry were calculated with the calibration of Tomkins *et al*.^53^ for inclusions in Type 1 garnet and intergrowths with Type 2 garnet (Table 2). Rutile is intergrown with quartz and is assumed to have grown in equilibrium with zircon. In the absence of zircon, the temperatures are considered as minimum estimates^53^. The precision on the estimates are 3–4%, encompassing the analytical uncertainty (~1% RSD) and a ±3% uncertainty in the calibration^53^. Ternary feldspar thermometry was applied to inclusions in Type 1 garnet, using calibrations at 0.8^54^ and 2.0 GPa^55^ (Fig. S1). The reintegration of mineral compositions was calculated based on proportions established from BSE images (*ImageJ*) and electron microprobe analysis. The temperature dependence of analysed Ti concentrations in Type 1 garnet provides an additional thermometer^39^. The thermometer was calibrated with and without orthopyroxene and therefore only provides approximate temperatures that match calibration curves. Neglecting exsolved Ti in rutile needles results in an underestimation of the garnet *T*.

Rock samples were measured for dry density using a Mettler Toledo AG-204 scale with density determination kit. Conservative uncertainties of 0.5% on the rock measurements are based on the variability of observed mineral modal abundance. Densities of modelled mineral assemblages were calculated internally within the THERMOCALC software, using the calcsv command^37^. Uncertainty on the calculations are likely to be <1% at the 2σ level^52^. Measured sample densities correlated with predicted values suggesting that the effects of expansion or contraction at *P–T* were within error.

## Electronic supplementary material

Supplementary Figure S1
