# Supplementary material for: Evaluating the importance of metamorphism in the foundering of continental crust
Source: Sci Rep. 2017 Oct 12;7:13039. doi: 10.1038/s41598-017-13221-6 (PMC5638824; doi:10.1038/s41598-017-13221-6)
Supplement: Supplementary file 1 — Supplementary Figure S1 [file 41598_2017_13221_MOESM1_ESM.pdf]

Evaluating the importance of metamorphism in the foundering of continental crust

Timothy Chapman, Geoffrey L. Clarke, Sandra Piazolo and Nathan R. Daczko

Supplementary Information

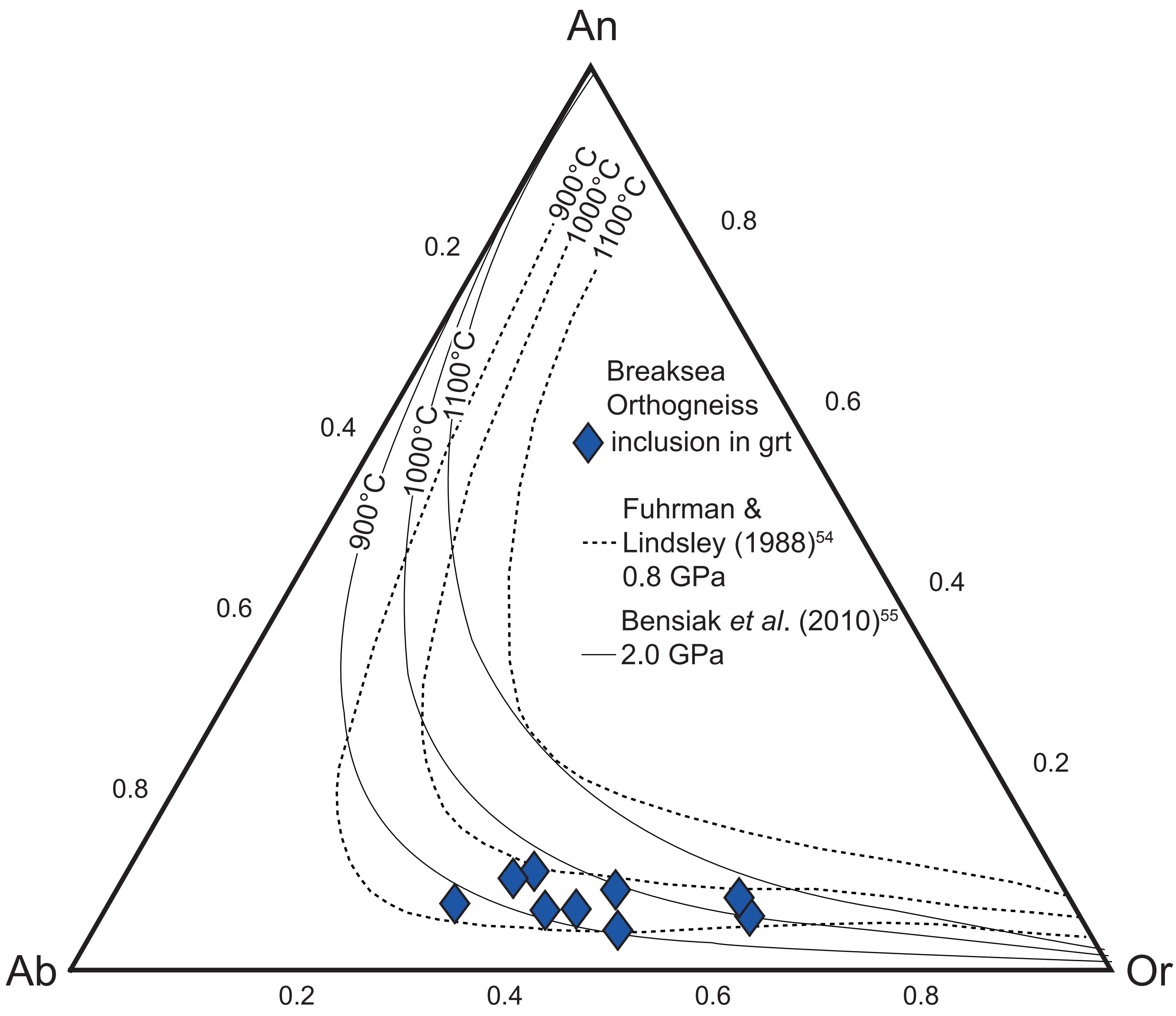

Supplementary Figure S1 An–Ab–Or ternary diagram of reintegrated feldspar inclusions in Type 1 garnet from the Breaksea Orthogneiss. Thermometry is based on isotherms of Fuhrman & Lindsay (1988)[54] at 0.8 GPa and Bensiak *et al*. (2008)[55] at 2.0 GPa.
